# Improvements in Iron Status and Cognitive Function in Young Women Consuming Beef or Non-Beef Lunches

**DOI:** 10.3390/nu6010090

**Published:** 2013-12-27

**Authors:** Cynthia Blanton

**Affiliations:** Dietetic Programs, Idaho State University, Pocatello, ID 83209, USA; E-Mail: blancynt@isu.edu; Tel.: +1-208-282-3953; Fax: +1-208-282-3937

**Keywords:** body iron, cognition, women, beef

## Abstract

Iron status is associated with cognitive performance and intervention trials show that iron supplementation improves mental function in iron-deficient adults. However, no studies have tested the efficacy of naturally iron-rich food in this context. This investigation measured the hematologic and cognitive responses to moderate beef consumption in young women. Participants (*n =* 43; age 21.1 ± 0.4 years) were randomly assigned to a beef or non-beef protein lunch group [3-oz (85 g), 3 times weekly] for 16 weeks. Blood was sampled at baseline, and weeks 8 and 16, and cognitive performance was measured at baseline and week 16. Body iron increased in both lunch groups (*p <* 0.0001), with greater improvement demonstrated in women with lower baseline body iron (*p <* 0.0001). Body iron had significant beneficial effects on spatial working memory and planning speed (*p <* 0.05), and ferritin responders (*n =* 17) *vs*. non-responders (*n =* 26) showed significantly greater improvements in planning speed, spatial working memory strategy, and attention (*p <* 0.05). Lunch group had neither significant interactions with iron status nor consistent main effects on test performance. These findings support a relationship between iron status and cognition, but do not show a particular benefit of beef over non-beef protein consumption on either measure in young women.

## 1. Introduction

Suboptimal iron status negatively impacts cognitive function in women of reproductive age, representing a significant health problem in light of the prevalence of iron deficiency. National surveys within North America report rates of iron deficiency in women aged 20–49 years of 15% (United States) [1], 19%–27% (Mexico) [2], and 9% (Canada) [3]. An even higher rate of iron deficiency among female university students in the United States (30%–50%) [4,5,6,7] poses the risk of compromised academic achievement due to deficiency-related cognition impairment.

In our recent study [8], of 42 female college students with varying levels of body iron, women with lower *vs*. higher body iron status took significantly longer to strategize their movements on a test of planning and working memory. This study was the first known to demonstrate that iron deficiency without anemia can impair cognitive function in adults. These findings are in agreement with those from observational and intervention studies performed on women of reproductive age [9,10,11,12,13,14,15]. Important to demonstrating causality, iron supplementation trials show parallel improvements in hematologic and cognitive variables [9,11,12,14].

While iron supplementation is an inexpensive means to treat iron deficiency, it is associated with side effects that compromise compliance [16,17,18]. The current project used a whole-food approach in correcting the cognitive impairment associated with iron deficiency. Observational studies and intervention trials support the benefit of increased meat intake in maintaining higher body iron status in premenopausal women [7,19,20], but no known investigations have examined the effect of beef consumption on cognitive performance in iron-deficient women. The present study tested the hypothesis that moderate consumption (here defined as 3 oz, 3 times weekly) of beef improves iron status and cognitive function in young women. Moderate intake of beef, a popular source of bioavailable iron, was selected as a reasonable dietary intervention that could be readily adopted by women. The control treatment of various non-beef foods contained levels of kcals and protein similar to those provided by the beef lunches.

## 2. Experimental Section

### 2.1. Study Design

The study was a 16-week prospective, randomized, controlled intervention trial conducted on the campus of Idaho State University. The study followed guidelines stated in the Declaration of Helsinki and all procedures involving human subjects were approved by the university’s Institutional Review Board of the Office of Human Research Protection. Written informed consent was obtained from all participants. Eligible female college students underwent baseline measures of iron status and cognitive function followed by 16 weeks of either beef or non-beef lunches consumed three times per week. Iron status assessment was repeated at midpoint and at endpoint, when cognitive function was tested a second time.

### 2.2. Participants

Women who were either currently enrolled at Idaho State University as an undergraduate or had recently (within past 6 months) graduated from the university were recruited using printed and electronic advertisements. Exclusion criteria included: age <18 or >30 years, body mass index <18 or >30 kg/m^2^, dietary exclusion of meat, current pregnancy or pregnancy within the previous year, current lactation, hormonal contraceptive use, irregular menses, smoking, regular high-intensity exercise level, current blood donation, dieting for weight loss, recent history of eating disorders, inflammatory or endocrine disorders, chronic inflammation [C-reactive protein (CRP) ≥ 3 mg/L], not fluent in the English language, current use of iron supplements, and excess alcohol consumption or use of recreational drugs, prescription drugs or herbal preparations that could interfere with iron absorption and/or affect mental performance. Women were selected for this study because suboptimal iron status is more common in women *vs*. men [10]. Undergraduate students at the same university were selected to reduce the effect of education level on cognitive performance scores. Eligible volunteers were non-obese because obesity is associated with altered iron status [21]. Volunteers not taking hormonal contraceptive agents were selected so that menstrual cycle phases could be monitored. This was necessary for scheduling of blood analysis and cognitive testing during the luteal phase of the menstrual cycle in order to control for cycle-related changes in cognitive function [22] and hematology [23]. Volunteers responding to advertisements completed a telephone screening and those meeting screening criteria were scheduled for an in-person appointment where study procedures were explained and informed consent obtained. Women were enrolled continuously over 18 months with both lunch groups running in parallel.

#### 2.1.1. Data Collection

##### 2.1.1.1. Anthropometrics

Height and weight were measured at baseline and weight measurements were repeated on weeks 8 and 17. Height was measured to the nearest 0.1 cm using a stadiometer. Weight was measured to the nearest 0.1 kg using a mechanical beam scale when volunteers were 12 h fasted and wearing light clothing and no shoes.

##### 2.1.1.2. Blood Analyses (Iron Assessment, Lipids, C-Reactive Protein)

Morning blood samples were obtained by antecubital venipuncture at baseline and weeks 8 and 17 from 12 h fasted volunteers during the luteal phase of their menstrual cycle. Blood was analyzed for complete blood count [(CBC), including red blood cell count (RBC), hemoglobin (Hb), hematocrit (Hct), mean corpuscular volume (MCV), mean corpuscular hemoglobin (MCH), and mean corpuscular hemoglobin concentration (MCHC)], serum iron, serum ferritin, serum soluble transferrin receptor (TfR), transferrin (Tf), and transferrin saturation. The CBC was measured using a Cell Dyn Sapphire Hematology System (Abbott Diagnostics, Santa Clara, CA, USA). Serum iron and serum ferritin were measured by a COBAS 6000 Clinical Chemistry Analyzer (Roche Diagnostics, Basel, Switzerland) using a colorimetric and electrochemiluminescent method, respectively. Serum Tf and TfR were measured by a Roche Modular Clinical Chemistry Analyzer (Roche Diagnostics, Basel, Switzerland) using an immunoturbidimetric assay. Body iron (mg/kg body weight) was calculated as: −[log(TfR/ferritin) − 2.8229]/0.1207 [24].

At baseline and week 17 serum lipids were measured to monitor the effects of the lunch intervention. Serum triglycerides and total and high-density lipoprotein (HDL) cholesterol were measured by a COBAS 6000 Clinical Chemistry Analyzer using an enzymatic, colorimetric method. Serum low-density lipoprotein (LDL) and very low-density lipoprotein (VLDL) concentrations were calculated from measured cholesterol and triglycerides values using the Friedewald equation [25]. Serum CRP was measured by particle-enhanced immunoturbidimetric assay using a COBAS 6000 Clinical Chemistry Analyzer (Roche Diagnostics). Serum CRP was used to screen for chronic inflammation, which can alter measures of iron status [26] and a level of 3.0 mg/L was set as the maximum cut-off for normal [27]. The experimental protocol stipulated that women with CRP levels ≥3.0 mg/L be excluded from continuing with the study; however, no participant met this criterion. 

##### 2.1.1.3. Assessment of Cognitive Performance

Cognitive performance was assessed at baseline and week 17. Immediately following blood sampling, participants consumed a controlled snack of 18 g whole-grain crackers, 55 g 2%-fat cottage cheese, and 500 mL bottled water. The snack served as a standardized countermeasure to the negative effects of fasting on cognitive function [28]. Thirty min following snack consumption, participants began the cognitive test session. Each participant was tested individually, in a session lasting approximately 40 min, inside a quiet, private room. One research staff member (the principal investigator) trained in administering the standardized cognitive battery and blinded to participants’ iron status conducted all testing. 

Cognitive function was assessed using the Cambridge Neuropsychological Test Automated Battery (CANTAB^®^) for Windows running on a 15.6 inch touch-screen tablet computer. The study used five CANTABeclipse version 3.0 tests administered in the following order: Motor Screening Test, Verbal Recognition Memory, One Touch Stockings of Cambridge, Spatial Working Memory, and Rapid Visual Information Processing. Participant responses were recorded by the touch screen for all tests except RVP, which utilized a press pad connected to the computer. The tests are described below. 

Motor Screening Test (MOT): This test is used to train the participant in pointing accurately. By measuring the speed and accuracy of pointing, it also serves as an index of motor skill. In the test, a series of crosses appears at different locations on the screen. Following a demonstration of touching the cross with the forefinger tip of the dominant hand, the volunteer points to ten crosses presented sequentially. The two outcome measures for the MOT are mean latency to touch the cross after it appears (in milliseconds) and mean error, which is the distance between the center of the cross and the location touched. The distance is measured in pixel units based on a screen resolution of 640 × 350 pixels.

Verbal Recognition Memory (VRM): This task assesses immediate and delayed memory of verbal information under conditions of free recall and forced choice recognition. The participant is shown a sequence of 12 words and is asked to: (1) verbally recall as many words as possible immediately after the presentation and (2) recognize the words she has seen before from a list of 24 words comprised of the original 12 and 12 distractors. After a 20-min delay, the participant is again asked to recognize the words she saw before from a list of the original 12 and 12 new distractors. The outcome measures are: free recall total correct, recognition total correct, and recognition total false positives (distractors).

One Touch Stockings of Cambridge (OTS): This task is based on the Tower of London test and assesses spatial planning ability and working memory. The participant is shown two displays of colored balls, one display at the top of the screen and one at the bottom. Each display shows three balls that appear to be suspended in a row of three stockings. Each stocking can accommodate up to three stacked balls. The goal is to move the balls in the bottom display so that they copy the pattern shown in the top display.

The participant is shown a demonstration of how to move one ball at a time by touching it and then touching the destination. The participant follows by solving three problems of increasing difficulty. Next, the participant is shown more problems that require her to calculate in her head the minimum number of moves needed to make the bottom display replicate the top display. The participant touches the appropriate box at the far bottom of the screen to indicate the minimum number of moves required. The four outcome measures are: problems solved on first choice, mean choices to correct, mean latency (in milliseconds) to first choice, and mean latency to correct.

Spatial Working Memory (SWM): This is a test of the ability to retain spatial information and to use working memory to manipulate remembered items. The test also assesses the ability to devise a strategy for solving the search task. A trial begins with multiple colored boxes displayed on the screen. The objective is to touch each box in turn until one opens with a blue token inside. Once found, a token is deposited in an empty column on the side of the screen. The process is repeated until a token has been found in each box and the column is full of tokens. The number of boxes is increased from 4 to 6 to 8 across the trials. Outcome measures are: errors (number of times the volunteer revisits a box in which a token has already been found), strategy (whether the volunteer follows a consistent pattern when searching for a token; a low score represents good strategy), mean latency (in milliseconds) to first response, mean time between box touches, and mean time to last response. 

Rapid Visual Processing (RVP): This is a test of sustained attention with a minor working memory component. A sequence of digits, ranging from 2 to 9, appears in a white box in the center of the computer screen. The digits are presented in pseudo-random order at a rate of 100 digits per minute and the entire task lasts 4 min. The aim is to detect consecutive odd or even target sequences and respond by touching the press pad. Target sequences appear 16 times every 2 min. The test is divided into 7 blocks: 1–4 (practice) and 5–7 (assessment). The primary outcome measures are: total hits (number of times the volunteer correctly responds within 1800 ms after the final digit of a sequence appears), total misses (number of times the volunteer fails to respond to a target sequence within the time frame), total false alarms (number of times the participant responds outside the time frame), total correct rejections (number of times the participant did not respond to non-target sequences), and mean latency to respond to target sequences within the 1800-ms time frame.

##### 2.1.1.4. Dietary Intervention

Women were randomly assigned to a beef or non-beef lunch group. Three lunches per week for 16 weeks were prepared and provided to the participants. The 4-month duration was chosen to allow sufficient time for brain iron levels to be replenished in iron-deficient women. Evidence indicates that 12 weeks of iron supplementation or consumption of a high-iron diet raises iron status measures [20]; however, data also show that liver iron concentrations are restored at a faster rate than brain iron levels [29]. Therefore, an 16-week intervention was deemed adequate in duration to detect an effect of iron intake on cognition. Two lunches per week (following a Monday and Wednesday or Tuesday and Thursday schedule for each woman) were consumed by participants in the university Dietetics Food Laboratory, and the third lunch was provided to participants for consumption at home over the weekend. Lunches followed a 4-week cycle menu and consisted of 3 oz (85 g) beef or non-beef entrée + 2 oz (56 g) starch + 8 oz (237 mL) bottled water (Table 1). Within each lunch day, the starch food was the same for all women and the beef or non-beef entrée was the same within each lunch group. Women were served lunch individually, at private tables between 11:00 a.m. and 1:00 p.m. Participants were observed during lunch to confirm complete consumption of the meal and that no other food or drink were consumed during the research lunch. Women confirmed consumption of the weekend lunch when returning the empty lunch bag the following week. To reduce confounding by the intake of beef outside the study, all women were instructed to not consume beef at other (non-study) meals more than once every other week.

**Table 1:** Nutrition information for intervention lunches ^1^.

Lunch type			
Beef, 3 oz/85 g	Kcal	Fe (mg)	Protein (g)
eye round roast	138	2.17	24.40
top sirloin	160	1.76	25.75
roast beef sliced	162	2.14	22.45
ground beef 90% lean	173	2.35	21.43
pot roast	173	3.00	26.38
beef short loin	163	1.65	24.57
Non-Beef, 3 oz/85 g			
egg substitute	40	1.66	8.40
marinated chicken breast	142	0.89	26.68
sliced turkey breast	88	1.22	14.50
cheddar cheese, low fat	343	0.58	28.14
ground turkey 93% lean	181	1.33	23.04
pork tenderloin	151	0.98	22.24
Ham	91	0.48	14.10
Turkey tenderloin, Foster Farms	90	1.40	21.00
Swiss cheese	323	0.17	22.90
Starch, 2 oz/56 g			
pasta	88	0.72	3.25
roll	174	2.08	6.08
white bread sandwich	148	2.01	5.12
small red potatoes	50	0.39	1.29
rice white, instant	66	0.99	1.22
hamburger bun	158	1.94	5.60
flour tortilla	161	1.12	4.88
Average per lunch			
Beef	282	3.50	28.08
Non-Beef	282	2.29	24.03

^1^ Each lunch consisted of one 3-oz portion beef or non-beef entrée + one 2-oz portion starch + water to drink. Nutrient information obtained from the United States Department of Agriculture National Nutrient Database for standard reference, release 26 [30].

##### 2.1.1.5. Assessment of Dietary Intake

Total dietary intake was assessed to monitor volunteer adherence to the study protocol and to investigate food and nutrient relationships with iron status. A computerized food-frequency questionnaire (FFQ; Block 2005 questionnaire with heme analysis, NutritionQuest, Berkeley, CA, USA) was self-administered by participants at five time points: baseline, week 5, week 9, week 13, and week 17. The questionnaire includes 110 food items and assesses nutrient intake for the past month. The food list was derived from NHANES 1999–2002 dietary recall data and the nutrient database was developed from the USDA Food and Nutrient Database for Dietary Studies, version 1.0 [31]. Individual portion size is asked for each food, and pictures are provided to enhance accuracy of quantification. Women were instructed to report intake during the past 30 days within each monthly FFQ and to include study lunches in their answers.

##### 2.1.1.6. Assessment of Covariates

Menstrual cycle phase, duration, and rate of flow were monitored using a self-administered paper questionnaire. Information on cycle phase was used to schedule blood draws and cognitive testing during the luteal phase and thereby minimize the confounding effects of cycle phase on hematology and cognitive performance [22,23]. Cycle duration and flow intensity data were included as covariates when analyzing hematology measurements across time.

#### 2.1.2. Statistical Analyses

Statistical analyses were performed using Statistical Analysis System (SAS) Enterprise Guide 4.3 running on SAS version 9.2 [32]. Blood analytes, cognitive test scores, and diet variables were tested for normality of distribution using the Kolmogorov-Smirnov test with the Lilliefors correction. Natural log-transformation was applied to non-normally distributed data prior to analyses. Mixed models analysis with Tukey’s correction for multiple comparisons was used to examine the effect of lunch group on iron status and cognitive test scores. For the OTS task, the dependent variable was time, the between-subjects effect was lunch group, and the within-subjects effects were body iron, move category (1–6), and session (baseline and endpoint). For the RVP and SWM tasks, analyses followed the same structure as that for the OTS task, except block (1–7) and box number (4,6,8), respectively, replaced move category. Analyses of the other cognitive tests followed the same structure except without the effect of move or block repetitions. Covariates of baseline iron status, menstrual cycle duration (in days) and menstrual cycle flow (days of moderate-heavy flow) were included in the model, but menstrual factors were removed when no effects were seen. Dietary data were examined by ANOVA and correlational analysis for effects on iron status measures. Absolute and adjusted [per 1000 kcals (4.184 MJ)] nutrient intakes were compared between lunch groups at baseline (FFQ 1) and during the intervention (FFQs 2–5). Intake measures across food-frequency questionnaires 2–5 did not differ significantly and therefore the mean intakes for each participant were calculated and used in analyses. Spearman’s rank correlation coefficient is reported for tests of correlation. Differences were considered significant at *p <* 0.05 and quoted levels are two-sided. Baseline hematologic and cognitive test data from 54 women who began but did not complete the study were analyzed as above, except without the variables of lunch group and repeated time points. 

Data were also examined for ferritin responders and non-responders to either lunch intervention. These analyses did not include lunch group as a variable. Differential responses to iron therapy occur [14,33,34] and a similar effect on iron status might be seen during a dietary intervention. Using the approach of Murray-Kolb and Beard [14], each participant was classified as a responder or non-responder based on whether she demonstrated a change in serum ferritin that was greater than or less than the published day-to-day variation (27%) in the circulating levels [35,36,37]. Published data on day-to-day variability in body iron measures are not known to exist, thus responder class was not based on body iron. Repeated measures Mixed Models ANOVA with Tukey’s correction were used to analyze the effect of response classification on change (endpoint–baseline) in cognitive test measures. 

## 3. Results

### 3.1. Demographics and Baseline Measurements

**Figure 1:**
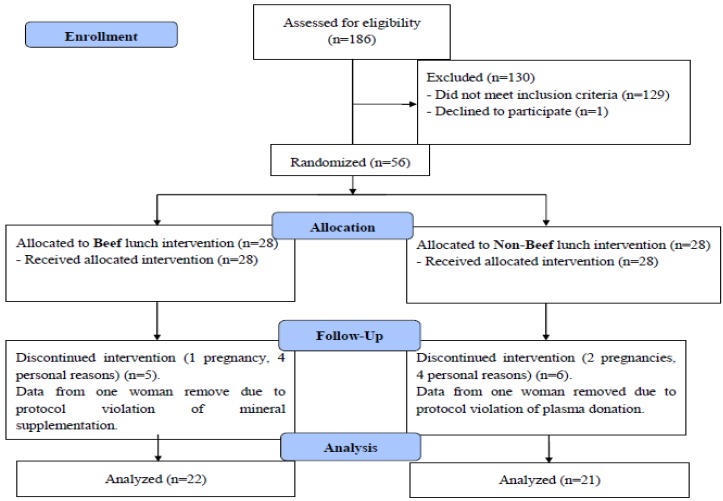
Volunteer flow diagram.

Figure 1 depicts the flow of participants through the study. Most of the 43 women who completed the study were of Caucasian race (*n =* 37), with the remaining being of mixed (*n =* 2 in each lunch group) or Latino (*n =* 1 in each lunch group) descent. At baseline, mean BMI, age, menstrual cycle duration and flow intensity, and measures of iron status and blood lipids were not significantly different between the beef and non-beef lunch groups (Table 2 and Table 3). Baseline body iron ranged from −2.73 to 11.64 mg/kg in the group of 43 women and was negative for one and three women in the beef and non-beef group, respectively. Four women in the beef group and 6 women in the non-beef group had abnormal serum ferritin (<14 ng/mL) and/or high TfR (>4.4 mg/L) at baseline. Baseline Hb was below the altitude-adjusted cutoff of 123 g/L [38] for 2 and 3 women in the beef and non-beef groups, respectively.

**Table 2:** Baseline characteristics of intervention participants.

Variable	All Women (*n* = 43)	Beef (*n* = 22)	Non-beef (*n* = 21)
Age (years)	21.14 ± 0.38	21.70 ± 0.62	20.56 ± 0.43
Body weight (kg)	64.80 ± 1.52	64.43 ± 2.36	65.18 ± 1.98
BMI (kg/m^2^)	23.27 ± 0.51	23.76 ± 0.77	22.76 ± 0.67

Values are means ± SE.

**Table 3:** Hematology and iron status measures (*n =* 43).

Variable *	Baseline		Midpoint (week 8)		Endpoint (week 16)		Absolute change (endpoint-baseline)		*P* value Main effect time	*P* value Main effect baseline measure	*P* value time × baseline measure
	Beef	Non-beef	Beef	Non-beef	Beef	Non-beef	Beef	Non-beef			
Hb (g/L) †	143.4 ± 2.4	138.5 ± 0.3	145.6 ± 0.2	141.5 ± 0.2	146.2 ± 0.2	140.7 ± 0.2	2.8 ± 1.8	2.1 ± 1.9	<0.0001	<0.0001	<0.0001
Hct (%)	41.6 ± 0.7	40.2 ± 0.7	42.5 ± 0.6	40.8 ± 0.5	42.4 ± 0.5	40.9 ± 0.5	0.8 ± 0.5	0.8 ± 0.7	<0.0001	<0.0001	<0.0001
RBC (millions/mm^3^)	4.7 ± 0.1	4.7 ± 0.1	4.8 ± 0.1	4.7 ± 0.1	4.8 ± 0.1	4.7 ± 0.1	0.1 ± 0.1	0.0 ± 0.1	<0.0001	<0.0001	<0.0001
MCV (µm^3^)	88.2 ± 0.7	87.1 ± 1.1	88.2 ± 0.8	86.9 ± 1.0	88.1 ± 0.8	87.3 ± 1.0	−0.1 ± 0.3	0.2 ± 0.6	<0.05	<0.0001	<0.05
MCH (pg)	30.4 ± 0.3	30.0 ± 0.5	30.3 ± 0.4	30.1 ± 0.5	30.4 ± 0.4	30.1 ± 0.5	0.0 ± 0.2	0.0 ± 0.3	NS	<0.0001	NS
MCHC (%)	34.5 ± 0.2	34.4 ± 0.2	34.3 ± 0.3	34.6 ± 0.2	34.5 ± 0.2	34.4 ± 0.3	0.0 ± 0.2	0.0 ± 0.3	<0.05	<0.0001	<0.05
Body iron (mg/kg) ††	6.5 ± 0.6	5.2 ± 0.8	6.5 ± 0.7	5.0 ± 3.8	7.0 ± 0.6	6.0 ± 0.5	0.5 ± 0.4	0.9 ± 0.5	<0.001	<0.0001	<0.0001
Serum ferritin (ng/mL)	33.6 ± 4.8	28.3 ± 4.2	34.0 ± 4.8	28.5 ± 4.6	41.6 ± 6.8	29.2 ± 3.0	8.0 ± 3.9	0.9 ± 2.7	<0.0001	<0.0001	<0.0001
TfR (mg/L) † ††	3.1 ± 0.2	3.8 ± 0.4	3.1 ± 0.2	3.8 ± 0.3	3.2 ± 0.2	3.4 ± 0.2	0.1 ± 0.1	−0.4 ± 0.2	<0.0001	<0.0001	<0.0001
Serum iron (µg/L)	1027.8 ± 89.1	1034.7 ± 115.8	921.8 ± 57.6	915.2 ± 94.0	955.5 ± 66.2	1064.8 ± 73.6	−72.0 ± 97.5	30.0 ± 112.9	<0.0001	<0.0001	<0.0001
Tf (mg/dL)	285.2 ± 9.0	290.0 ± 9.5	285.5 ± 9.6	297.4 ± 9.8	285.5 ± 10.5	288.3± 10.8	0.3 ± 6.2	−15.5 ± 16.7	NS	NS	NS
Tf saturation (%)	31.4 ± 3.3	31.5 ± 3.9	27.6 ± 2.4	26.7 ± 3.3	28.8 ± 2.5	31.1 ± 2.8	−2.6 ± 2.9	−0.4 ± 3.2	<0.0001	<0.0001	<0.0001

Beef group, *n =* 22; Non-beef group, *n =* 21. * Hb = hemoglobin; Hct = hematocrit; RBC = Red Blood Cell count; TfR = transferrin receptor; Tf = transferrin. Values are means ± SE. NS = Not statistically significant. Mixed Models ANOVA with repeated measures was used to determine main effects of time, lunch group, baseline measure, and interactions on baseline, midpoint, and endpoint blood measures; † Effect of lunch group, *p <* 0.05; †† Effect of lunch group × baseline measurement, *p <* 0.05.

### 3.2. Effects of Intervention and Baseline Iron Status on Change in Iron Status

There were significant effects of time, baseline measurement, and their interaction on all iron status and hematologic measures except Tf and MCH (Table 3). Lunch group had a significant main effect on TfR and Hb and the interaction of lunch group and baseline measurement was significant for body iron and TfR.

Post-hoc comparisons using the Tukey-Kramer adjustment showed no significant differences between lunch groups within time points for any iron status measure. For the group of 43 women, body iron and ferritin measures were significantly higher at endpoint *vs*. baseline and midpoint [body iron: *p =* 0.050 (endpoint *vs*. baseline) and *p =* 0.020 (endpoint *vs*. midpoint); ferritin: *p =* 0.042 (endpoint *vs*. baseline) and *p =* 0.021 (endpoint *vs*. midpoint)]. Differences between baseline and endpoint for Hb and Hct did not reach significance (*p =* 0.087, *p =* 0.088, respectively).

Baseline iron status had a significant effect on absolute change (endpoint-baseline) in iron status measures, such that women with lower *vs*. higher iron status displayed a greater magnitude of improvement in iron levels. Changes in body iron and ferritin were significantly affected by baseline levels (body iron: *p <* 0.0001); ferritin: *p <* 0.0001. There was a significant effect of lunch group (*p =* 0.0021), baseline TfR (*p <* 0.0001), and their interaction (*p =* 0.0005) on change in TfR. This interaction reflected a stronger relationship between baseline TfR and change in TfR in the non-beef *vs*. beef group: In correlational analysis, baseline TfR was significantly inversely correlated with change in TfR for the non-beef group (Spearman ρ = −0.66, *p =* 0.001) but not the beef group (ρ = −0.18, *p =* 0.42).

Seventeen women were classified as ferritin responders to either lunch intervention (Table 4). Responders *vs*. non-responders displayed a significantly greater percent increase in ferritin (*p <* 0.0001), percent decrease in TfR (*p =* 0.006), percent increase in Hb (*p =* 0.009), and absolute increase in body iron (*p <* 0.0001). The number of responders in the beef and non-beef groups was 10 and 7, respectively. There were no significant interactions between responder class and lunch group on iron status measures.

**Table 4:** Iron status measures in intervention women classified as ferritin responders or non-responders.

Variable *	Ferritin Responder (*n =* 17)	Ferritin Non-responder (*n =* 26)	*p* value
Ferritin, percent change	100.75 ± 17.51	−12.74 ± 4.08	*p <* 0.0001
TfR, percent change	−8.23 ± 3.69	5.90 ± 3.16	*p =* 0.006
Body iron, absolute change (mg/kg)	2.67 ± 0.36	−0.65 ± 0.24	*p <* 0.0001
Hb, percent change	5.23 ± 1.76	0.11 ± 0.98	*p =* 0.009

* Change between baseline and endpoint. Values are means ± SE. *P* values refer to between-group comparisons.

### 3.3. Effect of Intervention and Iron Status on Cognitive Test Performance

Verbal Recognition Memory (VRM): Lunch group and session had significant main effects on free recall of correct targets, with more words recalled by women in the beef *vs*. non-beef group (*p =* 0.007), and during the second *vs*. first session (*p =* 0.008). The higher word recall by women in the beef group was not statistically significant when compared within sessions (Tukey’s post-hoc, *p >* 0.05). Body iron and ferritin had significant negative effects on free recall women, with fewer words recalled by women with higher *vs*. lower body iron (*p =* 0.002) and ferritin (*p =* 0.001). Recognition of targets and distractors were not affected by lunch group, body iron, or session. 

Changes in VRM scores between ferritin responders *vs*. non-responders were not significantly different. 

One Touch Stockings of Cambridge (OTS): Mixed models ANOVA with repeated measures showed significant effects of body iron (*p =* 0.032) and ferritin (*p =* 0.015) on latency to correct choice for the higher-difficulty tasks (moves 4–6): Women with higher iron status required less time to make a correct choice. The effect of body iron (*p =* 0.058) but not ferritin (*p =* 0.024) was attenuated when all moves (1–6) were included in analyses. Mean number of choices to correct choice and number of problems solved on first choice were not significantly affected by body iron or ferritin. Lunch group did not have a significant effect on test measures.

**Figure 2:**
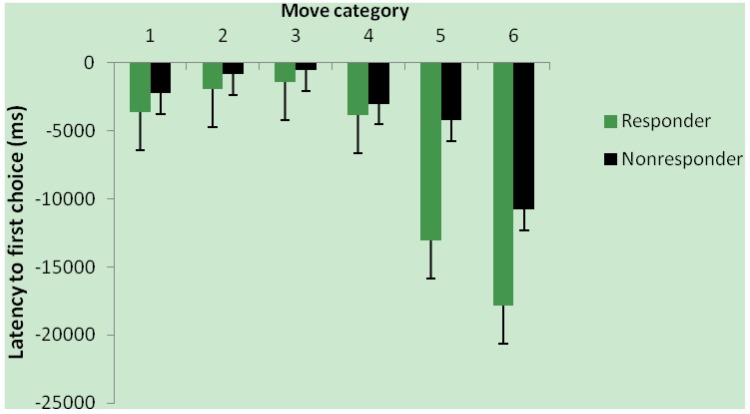
Change in response time by ferritin response group; Change (endpoint-baseline) in One-Touch Stockings of Cambridge latency to first choice for move categories 1–6 for ferritin responders (*n =* 17) and non-responders (*n =* 26). Main effect of responder group, *p =* 0.007.

Women classified as ferritin responders *vs*. non-responders showed a significantly greater improvement in latency to first choice between baseline and endpoint for moves 1–6 (*p =* 0.007; Figure 2). Mean number of choices to correct choice and number of problems solved on first choice were not significantly affected by responder class.

Spatial Working Memory (SWM): Latency to first response was significantly affected by body iron (*p =* 0.012), lunch group (*p =* 0.0003), box number (*p <* 0.0001), and session (*p =* 0.031): Greater speed was seen in women with higher *vs*. lower body iron, in the non-beef *vs*. beef group, in the less difficult (lower) box number trials, and in session 2 *vs*. 1. Token search time was significantly affected by body iron (*p =* 0.018), group (*p =* 0.003), box number (*p =* 0.008), and session (*p =* 0.001): Greater speed was demonstrated in women with higher body iron, in the non-beef group, in the lower box number tasks, and in session 2. Post-hoc analysis showed faster latency to first response and token search time in the non-beef group only in session 1 (*p <* 0.05). Women with higher *vs*. lower ferritin showed faster speed in latency to first response (*p =* 0.038) and token search time (*p =* 0.017). 

SWM strategy showed a significant effects of group (*p =* 0.018), box number (*p <* 0.0001) and session (*p =* 0.048), with better strategy demonstrated in women in the non-beef *vs*. beef group, in trials with fewer box numbers, and during the session 2 *vs*. 1. Post-hoc analysis showed no significant differences between groups within sessions, (*p >* 0.05). No main effects of body iron or ferritin on strategy were seen. Error scores showed no effect of body iron, ferritin, or group.

**Figure 3:**
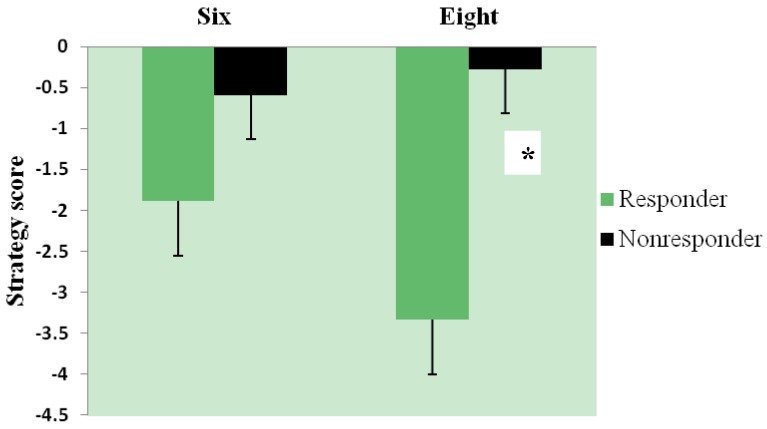
Change in strategy by ferritin response group; change (endpoint-baseline) in Spatial Working Memory strategy score for six- and eight-box problems in ferritin responders (*n =* 17) and non-responders (*n =* 26). A lower score and larger negative change indicate better strategy. Main effect of responder group, *p =* 0.007; * *p <* 0.05 between-group comparison for eight-box problem.

Ferritin responders demonstrated significantly greater improvement in strategy between baseline and endpoint than ferritin non-responders (*p =* 0.007, Figure 3).

Rapid Visual Processing (RVP): Latency to respond showed significant effects of ferritin (*p =* 0.023) and block (*p <* 0.0001): Faster speed was seen in women with higher *vs*. lower ferritin and in the earlier *vs*. later blocks. The effect of body iron on latency to respond approached but did not reach significance (*p =* 0.09). Neither lunch group nor session had significant effects on latency to respond.

Lunch group, session, and block had significant main effects on total hits: More hits were achieved in the beef *vs*. non-beef group (*p =* 0.0038) and during session 2 *vs*. 1 (*p <* 0.0001) and fewer hits were achieved as blocks progressed from 5 to 7 (*p =* 0.043). Consistently, total misses were significantly lower in the beef *vs*. non-beef group (*p =* 0.006) and in session 2 *vs*. 1 (*p <* 0.0001). Correct rejections were significantly higher in the beef *vs*. non-beef group (*p =* 0.009) and in session 2 *vs*. session 1 (*p <* 0.0001). The higher number of hits and correct rejections and lower number of misses by women in the beef *vs*. non-beef group was not statistically significant when compared within sessions (*p >* 0.05). Neither body iron nor ferritin had a significant main effect on RVP hits, misses, or correct rejections.

Ferritin responders *vs*. non-responders tended to show greater improvement in correct rejections (*p =* 0.056). No other effects on change in RVP scores were seen for responder class.

### 3.4. Relationship of Iron Status and Cognitive Function in All Women (*n* = 54) with Baseline Measures

Baseline demographic and iron status data from the larger group of 54 women who began the study were similar to those from the group of 43 women who completed the intervention (Table 5). None of the 11 women who did not complete the study had below-normal iron status. Like the group of women who completed the study, the group inclusive of all women with baseline data displayed an effect of iron status on word recall, response speed, and spatial working memory.

**Table 5:** Baseline characteristics of all women enrolled.

Variable	Women (*n =* 54)
Age (years)	21.70 ± 0.41
Body weight (kg)	64.99 ± 1.30
BMI (kg/m^2^)	23.47 ± 0.44
Body iron (mg/kg)	6.20 ± 0.46
Ferritin (ng/mL)	32.93 ± 2.88
TfR (mg/L)	3.30 ± 0.17
Hb (g/L)	140.94 ± 1.49

Values are means ± SE.

VRM: There were significant main effects of body iron (*p =* 0.034) and ferritin (*p =* 0.026) on free recall of correct target words, with more words recalled by women with lower *vs*. higher iron status. Iron status did not have a significant effect on recognition of targets or distractors.

OTS: There was a significant main effect of body iron (*p =* 0.010) and ferritin (*p =* 0.020) on latency to first choice: Women with higher iron status demonstrated greater speed. Neither the number of problems solved on first choice nor the number of choices to correct showed significant effects of body iron or ferritin.

SWM: Faster response time was displayed in women with higher iron status: Effect of body iron and ferritin on latency to first response, *p =* 0.047 and *p =* 0.060, respectively. There was a tendency toward shorter token search time in women with higher iron status: Effect of body iron and ferritin, *p =* 0.051 and *p =* 0.066, respectively. Strategy scores were significantly better in women with higher iron status: Effect of body iron and ferritin, *p =* 0.003 and *p =* 0.005, respectively.

RVP: The effect of ferritin on latency to respond approached significance (*p =* 0.07). Otherwise, there were no significant effects of body iron or ferritin on RVP scores.

### 3.5. Effect of Intervention on Blood Lipids (*n* = 43)

There was no significant effect of lunch group or time on blood lipids; nor did lunch intervention have a significant effect on change in blood lipids between baseline and endpoint.

### 3.6. Dietary Assessment

Women underreported dietary intake according to a cut-off point of 1.2 for the plausible ratio of energy intake to calculated basal metabolic rate [39,40]: ratio = 0.94 ± 0.06 (FFQ 1) and 0.76 ± 0.02 (FFQs 2–5). Mean reported kcal intake was higher for FFQ 1 [1612 ± 137 kcal (6.745 ± 0.573 MJ)] than for FFQs 2–5 [1310 ± 40 kcal (5.481 ± 0.167 MJ)] (main effect of FFQ number, *p =* 0.0006). Mean absolute and adjusted macro- and micronutrient intakes did not differ significantly between lunch groups at baseline. Baseline adjusted iron intake was significantly correlated with body iron (ρ *=* 0.33, *p =* 0.015; *n =* 43). No correlations were seen between body iron and adjusted intake of meat iron, heme iron, zinc, vitamin C, or protein at baseline.

Women reported lower adjusted protein intake during the intervention compared to baseline (*p =* 0.004), and women in the non-beef reported lower intakes of adjusted heme iron and adjusted meat iron during the intervention compared to those in the beef group (*p <* 0.05; Table 6). Burger and steak intake frequency was significantly different between baseline and the intervention (*p <* 0.0001), with higher intakes reported by women in the beef group (adjusted *p <* 0.01) and lower intakes reported by women in the non-beef group (adjusted *p <* 0.05).

**Table 6:** Reported dietary intake for lunch groups.

Lunch Group	Baseline				Intervention			
	**Adj protein (g)**	**Adj iron (mg)**	**Adj heme iron (mg)**	**Adj meat iron (mg)**	**Adj protein (g)**	**Adj iron (mg)**	**Adj heme iron (mg)**	**Adj meat iron (mg)**
Beef	44 ± 2.1	7.3 ± 0.3	0.5 ± 0.1	1.0 ± 0.2	41 ± 1 *	7.3 ± 0.2	0.6 ± 0.0 †	1.2 ± 0.1 †
Non-Beef	45 ± 1.8	7.5 ± 0.4	0.5 ± 0.1	1.1 ± 0.1	40 ± 1 *	7.7 ± 0.2	0.3 ± 0.0	0.4 ± 0.0

Note: Food-frequency questionnaire (FFQ) data for beef and non-beef lunch group at baseline (FFQ 1) and during the intervention (FFQs 2–5). Adj = Adjusted value expressed as per 1000 kcals (4.184 MJ); * *p <* 0.01 main effect of FFQ number (baseline *vs*. intervention); † *p <* 0.05 main effect of group; *p <* 0.01 effect of interaction of group × FFQ number; *p <* 0.01 Tukey’s post-hoc comparison of beef *vs*. non-beef.

## 4. Discussion

This study tested the efficacy of moderate beef consumption in ameliorating cognitive impairment associated with low iron status. The current findings support a positive relationship between iron status and cognitive function, but they do not show that moderate intake of beef improves iron status or cognitive performance in women with decreased iron status to a greater degree than non-beef protein foods. Young women with higher *vs*. lower body iron and ferritin performed better on tests of planning speed and spatial working memory and women classified as ferritin responders *vs*. non-responders displayed more pronounced improvements in tests of planning speed, spatial working memory strategy, and attention following the intervention. 

These results replicate and extend findings from our prior observational investigation showing a significant relationship between planning speed and body iron in young college women [8]. Both studies tested women of similar age, BMI, and education level and found slowed performance on a task of planning ability (Stocking of Cambridge in the present study and Tower of London in the previous study) in women with lower body iron. The current study further reports a significant effect of iron status on response latencies in tests of spatial working memory and rapid visual processing, and search time and strategy in a test of spatial working memory. The results are consistent with those reported by Murray-Kolb and Beard [14], who found iron deficiency-related impairments in multiple domains of cognitive performance in college-educated women 18–35 years of age. These investigators showed that severity of iron depletion correlated with degree of cognitive impairment in three groups of women: iron-deficient anemic, iron-deficient non-anemic, and iron-sufficient. Following supplementation with 60 mg elemental iron/day for 16 weeks, ferritin responders *vs*. non-responders showed significant improvements in attention, learning, and memory task accuracy, but not task speed. Increased speed was seen only in women classified as Hb responders. The different findings between the present study and that of Murray-Kolb and Beard regarding ferritin response and improved task speed might be related to differences in participant population and treatment. The current study included fewer women with a more narrow range of iron status levels and a less potent iron dose. Regarding the finding of an inverse effect of iron status on verbal free recall in the present study, this is inconsistent with existing evidence of improved memory with higher iron status [41,42] and cannot be readily explained in the context of the overall results presented here. The absence of this effect in the present study’s ferritin responder and non-responder groups suggests the finding might be an artifact.

Other intervention studies of females of reproductive age demonstrate corrections in cognitive impairment and iron status in response to iron supplementation [9,11,12,13,43]. The doses of elemental iron used in these studies ranged from 18 to 195 mg/day and whether naturally iron-rich foods provide sufficient iron to induce similar responses is not known to have been reported prior to the present study. Dietary interventions using iron-fortified foods have shown efficacy in improving iron status in adults. In a 12-week intervention in young adult non-anemic women, Hoppe *et al*. [44] showed comparable increases in body iron and ferritin resulting from either consumption of blood-based crisp bread (35 mg iron, 27 mg being heme) or supplementation with iron (35 mg and 60 mg). Karl *et al*. [45] demonstrated that consumption of iron-fortified bars (2 per day, 27.9 mg iron each) protected iron-deficient anemic female soldiers against training-associated declines in iron status better than a control food. Using a lower level of fortification, Haas *et al*. [46] substituted high-iron rice for local rice for 9 months in a group of Filipino young women and showed a significant increase in body iron in non-anemic participants. This study demonstrated that a relatively small increase in dietary iron intake (1.41 mg/day increase above control) over an extended period can significantly improve iron status.

Meat-based interventions appear less frequently in the literature than supplementation and fortification studies, but their results indicate benefit in supporting iron levels in women. Lyle *et al*. [47] showed that a high food − iron + meat supplement diet protected iron status more effectively than iron supplementation in university women participating in an exercise program. Notably, total iron intake in the high food − iron + meat group was 11.8 ± 2.8 mg, significantly less than that in the 50 mg iron-supplemented group (57.8 ± 2.4 mg) The present study and that of Lyle *et al*. differ in several respects, yet both studies show improved iron status in women consuming ~1.5 servings of meat per day. Further, beef was shown to be superior to poultry and fish in improving serum ferritin in intervention studies of adolescents [48] and iron-deficient women [49]. Lastly, systematic reviews support the efficacy of dietary iron interventions for improving iron status. A meta-analysis by Casgrain *et al*. [50] reported that iron supplementation significantly improved iron status and that supplementation form (pills, meat, and fortified food) was not a significant modifier of ferritin or TfR response. Altogether, findings from the present and previous studies indicate that food-based approaches, including regular, moderate meat consumption, are beneficial in protecting and increasing iron nutriture. 

Parallel changes in blood iron levels and cognitive task performance are suggestive of a causal relationship between iron and brain function. Brain activity, measured by electroencephalography (EEG), is altered as a function of iron status. Wenger *et al*. [51,52] recently reported significant relationships between iron status, attention and memory abilities, and EEG patterns in adolescents and women. Correcting iron-deficiency anemia with iron supplementation can reverse EEG abnormalities [53] and the associated impairments in cognitive task performance [12]. These studies build on early findings by Tucker *et al*. [54,55] of associations between EEG patterns, cognitive ability, and iron status. The mechanisms underlying altered brain activity in relation to iron status are not fully understood but may involve disruptions in neuroendocrine function. Iron is a cofactor for enzymes synthesizing catecholamines, serotonin, and the thyroid hormones and alterations in their levels and activity are seen in iron-deficient humans and animals [56,57].

The unexpected finding of improved iron status in both lunch groups seems to suggest that regular (3 times weekly), nutrient-dense lunches improved the women’s diet quality enough to affect blood iron indicators. Women’s mean body weight increased 0.8 kg, which was not statistically significant (*p >* 0.9) but could indicate greater kcal and nutrient intake during the intervention. This possibility is difficult to substantiate since women underreported dietary intake and physical activity was not measured. Poor diet quality in college students is common [58,59,60] and the study lunches might have displaced less nutritious food normally consumed by the women in this intervention. The present study was designed to test the effect of the total nutrient package of beef, rather than the effect of a particular nutrient, on iron status and cognitive function. While adjusted intakes in the beef *vs*. non-beef protein groups were greater for heme iron, meat iron, and absolute servings of steak and hamburger, no differences between the groups were seen in adjusted protein and adjusted total iron intakes. The lack of observable differences in iron status between the lunch groups could also be related to the moderate level of beef intervention used in this study, which was chosen to simulate a reasonable dietary strategy. A specific benefit of consumption of beef over other foods on iron status might require larger intakes over a longer duration to observe. A two-fold higher incidence of negative iron balance has been observed in British women reporting no *vs*. high consumption of red meat [61].

Strengths of this study include a participant population of women similar in education level, age, physical activity level, and BMI who were tested for iron status and cognitive function during the same menstrual phase and under the same fasted (blood draw) and fed (controlled pre-cognitive testing snack) conditions. Also, the study lunches were prepared for the participants and two of every three lunches were consumed in the presence of research staff. Lastly, one research staff member blinded to participant iron status performed all cognitive tests.

Limitations include the homogeneous participant population, which restricts generalizability of results to other groups. Further, this study did not restrict enrollment to women with low iron status, which would have allowed for improvements in iron status to be observed more clearly. While the women were instructed on allowable beef intake outside the study and dietary intake was monitored by monthly FFQs, this factor was not strictly controlled in these free-living participants. Also, the women were not blinded to treatment allocation. The women’s experience with computerized games could have affected performance on the cognitive tests [62], but this was not measured. Lastly, a training effect of repeated cognitive testing is known to result in improved performance irrespective of intervening treatment. However, this effect was common across women and session was taken into account during data analysis.

## 5. Conclusions

In conclusion, the present study contributes to the body of evidence demonstrating a direct relationship between iron status and cognitive function in adult women. Further, the results demonstrate coincident improvements in iron status and planning speed, spatial working memory strategy, and attention in ferritin responders. A consistent, differential effect of moderate beef *vs*. non-beef protein consumption on iron status or cognitive function was not seen, and both types of lunches showed benefit in these measures in young college women.
